# Systematic reviews are rarely used to contextualise new results—a systematic review and meta-analysis of meta-research studies

**DOI:** 10.1186/s13643-022-02062-8

**Published:** 2022-09-05

**Authors:** Eva Draborg, Jane Andreasen, Birgitte Nørgaard, Carsten Bogh Juhl, Jennifer Yost, Klara Brunnhuber, Karen A. Robinson, Hans Lund

**Affiliations:** 1 Department of Public Health, University of Southern Denmark, Odense, Denmark; 2 Department of Physiotherapy and Occupational Therapy, Aalborg University Hospital, Denmark and Public Health and Epidemiology Group, Department of Health, Science and Technology, Aalborg University, Denmark, Aalborg, Denmark; 3 Department of Sports Science and Clinical Biomechanics, University of Southern Denmark and Department of Physiotherapy and Occupational Therapy, Copenhagen University Hospital, Herlev and Gentofte, Denmark; 4 M. Louise Fitzpatrick College of Nursing, Villanova University, Villanova, USA; 5 Digital Content Services, Elsevier, London, UK; 6 Johns Hopkins University School of Medicine, Baltimore, USA; 7 Section of Evidence-Based Practice, Western Norway University of Applied Sciences, Bergen, Norway

**Keywords:** Systematic review, Evidence-based research, Context

## Abstract

**Background:**

Results of new studies should be interpreted in the context of what is already known to compare results and build the state of the science. This systematic review and meta-analysis aimed to identify and synthesise results from meta-research studies examining if original studies within health use systematic reviews to place their results in the context of earlier, similar studies.

**Methods:**

We searched MEDLINE (OVID), EMBASE (OVID), and the Cochrane Methodology Register for meta-research studies reporting the use of systematic reviews to place results of original clinical studies in the context of existing studies. The primary outcome was the percentage of original studies included in the meta-research studies using systematic reviews or meta-analyses placing new results in the context of existing studies. Two reviewers independently performed screening and data extraction. Data were synthesised using narrative synthesis and a random-effects meta-analysis was performed to estimate the mean proportion of original studies placing their results in the context of earlier studies. The protocol was registered in Open Science Framework.

**Results:**

We included 15 meta-research studies, representing 1724 original studies. The mean percentage of original studies within these meta-research studies placing their results in the context of existing studies was 30.7% (95% CI [23.8%, 37.6%], *I*^2^=87.4%). Only one of the meta-research studies integrated results in a meta-analysis, while four integrated their results within a systematic review; the remaining cited or referred to a systematic review. The results of this systematic review are characterised by a high degree of heterogeneity and should be interpreted cautiously.

**Conclusion:**

Our systematic review demonstrates a low rate of and great variability in using systematic reviews to place new results in the context of existing studies. On average, one third of the original studies contextualised their results. Improvement is still needed in researchers’ use of prior research systematically and transparently—also known as the use of an evidence-based research approach, to contribute to the accumulation of new evidence on which future studies should be based.

**Systematic review registration:**

Open Science registration number https://osf.io/8gkzu/

**Supplementary Information:**

The online version contains supplementary material available at 10.1186/s13643-022-02062-8.

## Background

The number of clinical health research studies is increasing rapidly, a trend that requires additional time and money resources and places greater demands on participants who are enrolled in these studies, potentially increasing the risk of harmful effects [1–4]. Therefore, a central question in research is ‘Does additional work add new knowledge, or does it confirm what we already know?’ While determining the answer to this question is of utmost importance when planning a new study, it is also critical to ask this question after finishing a study to establish its contribution to existing knowledge and demonstrate how it contributes to the cumulative evidence [5–7]. However, this is only possible when authors discuss their findings considering existing evidence. Given the benefits of existing evidence syntheses (e.g. systematic reviews [SR] with or without a meta-analysis [MA]), having already systematically and transparently synthesised existing knowledge, it follows that researchers should be conducting or referring to evidence synthesis relevant to the study topic in the ‘Discussion’ section of works publishing study results.

The use of existing knowledge systematically and transparently has been emphasised for years; it is a component of the CONSORT [8] and the QUOROM [9] statements in 1999 and has since become a requirement for publication in *The Lancet* in 2005, 2010 and 2014 [10–12] and a key issue for international organisations, such as the Reward Alliance (https://www.rewardalliance.net) and the Evidence-Based Research Network (https://evbres.eu) [2, 13, 14]. The latter of the two was established to reduce waste in research by promoting an evidence-based research (EBR) approach during all stages of the research process, stating, ‘For scientific, ethical and economic reasons, current high-quality systematic reviews need to be seen as an essential component of decisions about [ …….] the interpretation of new study results’ [2].

This SR and MA aimed to identify and synthesise results from meta-research studies examining if and how original clinical studies use SRs to place their results in the context of earlier studies. No other SRs of meta-research studies with similar aims has been uncovered in the existing literature.

## Methods

Prior to the study, the protocol was registered in Open Science Framework (OSF) (https://osf.io/8gkzu/) and remained unchanged during the review except for adjustments of risk of bias from 13 to 10 items and to solely focusing on the risk of bias, leaving out reporting quality. This review is reported in accordance with the Preferred Reporting Items for Systematic Review and Meta-Analysis (PRISMA) guidelines [15].

### Search strategy and selection criteria

This study is one of six evidence syntheses (five systematic reviews and one scoping review) conducted to assess the global state of EBR in clinical research. Given the common aim across the evidence syntheses, an overall search strategy was designed to identify meta-research studies assessing if researchers used (a) earlier similar studies and/or SRs of earlier similar studies to inform the justification and/or design of a new study, (b) SRs to the interpretation of new results or (c) meta-research studies to assess if redundant studies were published within a specific area.

The first search was performed in June 2015 and included MEDLINE via both PubMed and Ovid, EMBASE via Ovid, CINAHL via EBSCO, Web of Science (Science Citation Index Expanded [SCI-EXPANDED]), Social Sciences Citation Index (SSCI), Arts & Humanities Citation Index (A&HCI) and Cochrane Methodology Register (CMR, Methods Studies) from inception. Reference lists of the included studies were screened for relevant publications as well as authors’ personal libraries, and abstracts from the Cochrane Methodology Reviews were screened. No language or publication year restrictions were applied.

An updated search strategy was developed based on the initial search from 2015 and used in MEDLINE and Embase via Ovid from January 2015 to June 2021. Again, the reference lists of new included studies were screened for relevant references as well as authors’ personal libraries, and abstracts from January 2015 to June 2021 of Cochrane Methodology Reviews were screened. The full search is outlined in Additional file 1 and documented in the PRISMA-S Checklist in Additional file 2.

We included meta-research studies about clinical research (i.e. studies studying research on research) that reported findings on the use of SRs when placing new results in the context of earlier, similar clinical studies. Our definition of meta-research is grounded on Ioannidis’s definition of meta-research as ‘the study of research itself: its methods, reporting, reproducibility, evaluation and incentives’ [16]. To be included, the meta-research studies needed to examine the use of SRs in the ‘Discussion’ sections of original studies so it can be determined if they placed their results in the context of earlier, similar studies.

Search results were uploaded to Rayyan (https://rayyan.qcri.org/welcome) for screening, and duplicates were removed in Endnote.

### Data extraction and quality assessment

The search results from the first search (June 2015) were independently screened by 10 pairs of two reviewers, with each pair consisting of one reviewer with experience as a systematic reviewer and one with less experience. Both reviewers initially screened the same 50 publications and discussed the results to secure consistency in their assessments before beginning screening for the reviews. Disagreements on study selection were reached by consensus and discussion with a third reviewer (HL) if needed. Four reviewers (KR, KB, CB, HL) performed the full-text screening independently. This initial screening resulted in a gross list of meta-research studies relevant to all the abovementioned reviews and the scoping review.

**Fig. 1:**
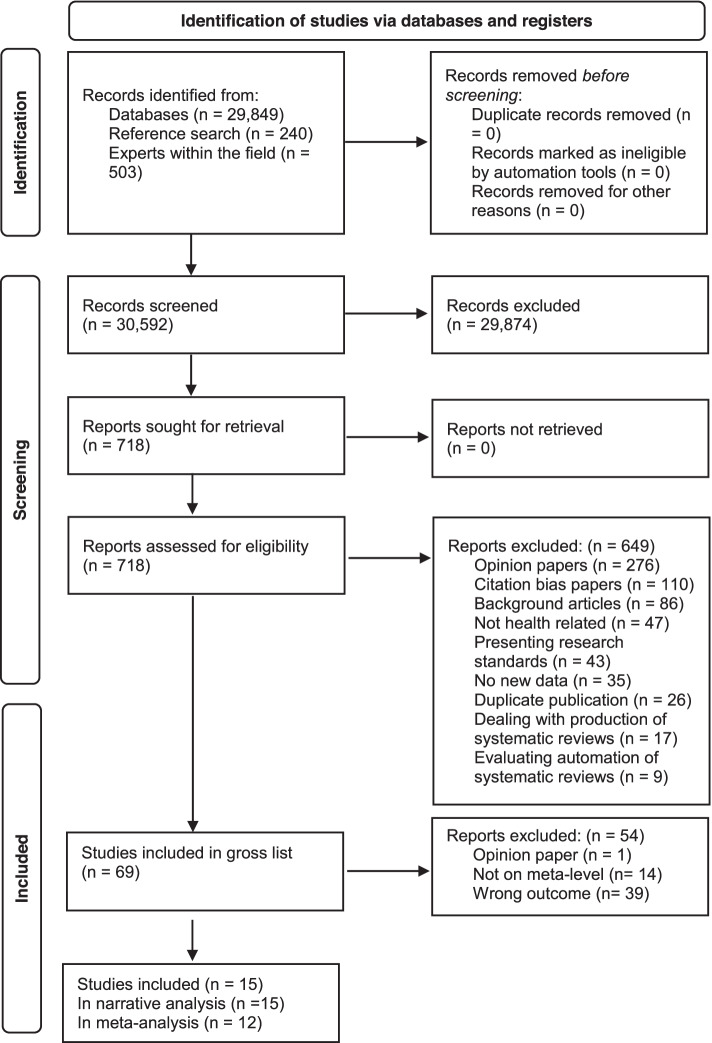
PRISMA flow diagram

Next, two reviewers (ED, JA) independently screened the titles and abstracts for this specific SR and applied the specific screening criteria for this study (i.e. contextualising new results with earlier SRs in the ‘Discussion’ section). Subsequently, the full text of all meta-research studies meeting the title and abstract criteria and categorised as potentially relevant was reviewed independently by the same two reviewers (ED, JA) using predetermined screening criteria with disagreements resolved through discussion and consensus. The study selection process is documented in the flowchart (Fig. 1).

We developed and pilot-tested a data extraction form to extract data for study characteristics and outcomes of interest. Two reviewers (ED, JA) independently extracted data, with a third reviewer (BN) available to resolve disagreements.

As a thorough search did not detect any standard tool available to assess the risk of bias of empirical meta-research studies, the Editorial Group of the Evidence-Based Research Network compiled a list of items considered important for assessing the risk of bias in meta-research studies. The list was tested on a sample of included meta-research studies, and following a discussion, the number and content of the list of items were adjusted. The final version included 10 items deemed *low risk of bias*, *high risk of bias* or *unclear risk of bias*. To ensure a rigorous and fair assessment, each item was added with one or two prompts to specify a high risk of bias (see Additional file 3). Applying this final version, each meta-research study meeting the inclusion criteria for this SR was appraised independently by two authors (ED, JA) to determine the risk of bias. Divergences were solved through discussion (BN, CJ, ED, HL, JA). No study was excluded due to low quality.

### Data analysis

The following outcomes were defined: percentage of original studies included in meta-research studies placing their results in the context of earlier, similar studies in the ‘Discussion’ sections (primary outcome); qualitative text analysis on how the meta-research studies placed their results in the context of SRs by choice of wording and phrasing; and percentage of original studies included in meta-research studies quantitatively integrating findings of the results of the earlier original studies by updating an SR and/or MA.

The following study characteristics were extracted from each of the included meta-research studies: bibliographic information, study aims, study design, material, country (based on the first author’s affiliation), inclusion period, area of interest, results, and conclusion. Further, the results in terms of the primary and secondary outcomes were extracted in duplicate by two reviewers (ED, JA).

The characteristics of the included meta-research studies, their risk of bias assessments and results across the original studies reported in the meta-research studies were narratively summarised. Furthermore, an MA using the random-effects model (DerSimonian and Laird) was used to determine the overall estimate and perform a forest plot of original studies using an SR to place their study in the context of earlier studies, as this model is the default when using the ‘metaprop’ command. Heterogeneity was assessed by estimating the *I*^2^ statistics, describing the percentage of variance attributable to inconsistency rather than the chance and the between-study variance tau^2^ [17]. When investigating reasons for heterogeneity, a restricted maximum likelihood (REML) method was used and covariates with the ability to reduce tau^2^ were deemed relevant. All analyses were performed in Stata, version 17.0 (StataCorp. 2019. *Stata Statistical Software: Release 17*. College Station, TX: StataCorp LLC).

## Results

The first broad search prompted 30,592 unique citations after the removal of duplicates, of which 29,874 were not included based on title and abstract screening. Of the 718 citations proceeding to full-text screening, 649 were not included, leaving 69 citations that met the inclusion criteria—of these, 15 were deemed relevant to this SR, representing 1724 original studies. For a list of included studies, please see Additional file 4. For a list of reasons for exclusion and further details, please see Fig. 1.

### Study characteristics

The earliest meta-research study was published in 1998 [18] and the most recent in 2021 [19] with 8 out of 15 published within the latest 5 years from 2017 to 2021 [19–26]. Two thirds of the meta-research studies originated from Europe—six from the UK [18, 27–31], one from Croatia [26], two from Germany [20, 32] and one from Switzerland [23]—and the remaining five meta-research studies originated from the USA [19, 21, 22, 24, 25]. All meta-research studies were cross-sectional studies of available evidence. The majority of meta-research studies narratively synthesised the available evidence with only one study synthesising the available evidence quantitatively using MA [26].

The meta-research studies were generally limited to including randomised studies published in a specific time in selected high-ranked journals (*n* = 13) [18, 19, 21–31]. Two meta-research studies deviated from this approach; one meta-research study examined MA included in a particular SR and MA [32] and one meta-research study was bounded to a sample of original studies from a specific database [20]. In terms of the clinical research area, nine meta-research studies stated a specific focus on anaesthesiology [26], pharmacological treatment [32], physiotherapy [20], orthopaedia [21], obstetrics and gynaecology [22], urology [19], ophthalmology and optometry [25], general medicine [24] or surgery [23], while the remaining six meta-research studies did not single out a specific speciality. The study by Hoderlein et al. [20] included two cohorts: one from 2001 and one from 2015.

Altogether, the 15 meta-research studies included in this SR assessed 1724 original studies, and the number of included original studies included in the individually meta-research studies varied from 18 [28] to 637 [24]. Nine studies included less than 100 original studies [18, 20, 23, 27–32] (Hoderlein et al. [20] Cohort 1), and the remaining six studies [19–22, 24–26] (Hoderlein et al. [20] Cohort 2) between 128 [21] and 637 original studies [24]. Details of meta-research study characteristics are presented in Table 1.

**Table 1:** Characteristics of included studies (*N*=15)

Study	Study aim	Study design	Material	Country	Inclusion period	Area of interests	Results	Conclusion
Clarke M, Alderson P, Chalmers I. (2002) [27]	To assess whether RCTs in 5 general medical journals discuss new results in light of available evidence.	Cross-sectional	RCTs published in *Annals of Int Med, BMJ, JAMA, Lancet* and *NEJM*	UK	May 2001	No specific speciality	33 RCTs included. 0 (0%) updated a SR. 3 (9%) cited SR	Little evidence exists to suggest the results of an RCT are discussed in light of the totality of the available evidence.
Clarke M, Chalmers I. (1998) [18]	To assess whether RCTs in 5 general medical journals discuss new results in light of available evidence.	Cross-sectional	RCTs published in *Annals of Int Med, BMJ, JAMA, Lancet* and *NEJM*	UK	May 1997	No specific speciality	26 RCTs included. 0 (0%) updated a SR. 6 (23%) cited SR	Little evidence exists to suggest journals have adequately implemented the CONSORT recommendation that results of an RCT be discussed in light of the totality of the available evidence.
Clarke M, Hopewell S. (2013) [30]	To assess whether RCTs in 5 general medical journals discuss new results in light of available evidence.	Cross-sectional	RCTs published in *Annals of Int Med, BMJ, JAMA, Lancet* and *NEJM*	UK	May 2012	No specific speciality	35 RCTs included. 2 (6%) updated an SR. 11 (31%) cited SR	Many trials still do not use SRs in their reporting.
Clarke M, Hopewell S, Chalmers I. (2007) [28]	To assess whether RCTs in 5 general medical journals discuss new results in light of available evidence.	Cross-sectional	RCTs published in *Annals of Int Med, BMJ, JAMA, Lancet* and *NEJM*	UK	May 2005	No specific speciality	18 RCTs included. 0 (0%) updated a SR. 5 (28%) cited SR	Little evidence suggests results of an RCT are discussed in light of the totality of the available evidence. Although the proportion of trials referring to SRs in ‘Discussion’ sections has increased, the majority of reports continued to fail even to do this.
Clarke M, Hopewell S, Chalmers I. (2010) [29]	To assess whether RCTs in 5 general medical journals discuss new results in light of available evidence.	Cross-sectional	RCTs published in *Annals of Int Med, BMJ, JAMA, Lancet* and *NEJM*	UK	May 2009	No specific speciality	29 RCTs included. 1 (4%) updated an SR. 10 (35%) cited SR	Little evidence exists to suggest results of an RCT are discussed in light of the totality of the available evidence. Although the proportion of trials referring to SRs has increased, most reports still fail to do this.
Engelking A, Cavar M, Puljak L. (2018) [26]	To analyse whether existing SRs were mentioned in RCTs published as a rationale for discussing the results.	Cross-sectional and meta-analysis	RCTs published in *Anaesthesia, Anaesthesia and Analgesia, Anaesthesiology, Pain, British Journal of Anaesthesia, European Journal of Anaesthesiology, Regional Anaesthesia* and *Pain Medicine*	Croatia	2014–2016	Anaesthesia, anaesthesia and analgesia, anaesthesiology, pain,	622 RCTs included. 245 (39%) cited SR	No conclusion regarding placing new results in contexts of earlier results in the discussion section
Goudie AC et al. (2010) [31]	To assess the extent to which authors currently make use of previous trial evidence in the reporting of RCTs.	Cross-sectional	RCTs published in JAMA and Archives of Internal Medicine	UK	January–May 2007	No specific speciality	27 RCTs included. 1 (4%) updated an SR. 10 (37%) cited SR	No conclusion regarding placing new results in contexts of earlier results in the discussion section
Helfer B et al. (2015) [32]	To assess whether recent meta-analyses cite, describe, and discuss previous meta-analyses and SRs.	Cross-sectional	Meta-analyses published in NEJM, Lancet, JAMA, Annals of Int Med, PLOS Medicine, BMJ (pharmacological treatment)	Germany	January 2012–March 2013	Pharmacological treatment	52 meta-analyses included. 25 (48%) cite SR	Meta-analyses on pharmacological treatments do not consistently discuss the findings of previous meta-analyses on the same topic.
Hoderlein X, Moseley AM, Elkins MR. (2017) [20]	To investigate the extent to which RCTs use high-quality clinical research to interpret the trial’s results and to assess a possible progress between 2001 and 2015.	Cross-sectional	Clinical trials randomly selected from Physiotherapy Evidence Database	Germany	2001 and 2015	Physiotherapy	2001: 70 RCTs included. 0 (0%) updated a SR. 12 (17%) cited SR. 2015: 151 RCTs included. 1 (1%) updated an SR. 52 (34%) cited SR	Citing is increasing from 2001 to 2015, but integration with existing research in the ‘Discussion’ section is rare.
Johnson AL, Walters C, Gray, H et al. (2020) [21]	To evaluate the use of SRs to justify RCTs…. And analyse the reference of SRs for trial justification in RCTs.	Cross-sectional	RCTs published in 3 high-ranking orthopaedic journals, and for comparison RCTs published in general orthopaedic journals	USA	January 1, 2015, to November 30, 2018	Orthopaedia	128 RCTs included. No data on SRs in the ‘Discussion’ section	SRs are frequently cited in orthopaedic trauma RCTs but are not commonly cited as justification for conducting a clinical trial.
Rauh S, Nigro T, Sims M et al. (2020) [22]	To analyse published articles for citation of SRs for justification of conducting RCTs.	Cross-sectional	RCTs in obstetrics and gynaecology journals	USA	January 1, 2014–December 31, 2017	Obstetrics and gynaecology	458 trials were included. 207 (45.2%) cited SRs	A large portion of the RCTs recently published in clinical obstetrics and gynaecology journals are not citing SRs as justification for conducting their studies, which may be leading to an increase in research waste.
Rosenthal R, Bucher HC, Dwan K. (2017) [23]	The aim was to investigate to what extent information from SRs is used to synthesise results.	Cross-sectional	RCTs published in *Annals of Surgery, JAMA Surgery, British Journal of Surgery*	Switzerland	2010	Surgery	51 RCTs included. 0 (0%) updated an SR. 9 (18%) cited SR	SRs are considered for summarising findings […] rather than for knowledge synthesis after trial conduct.
Shephard S, Wise A, Johnson BS et al. (2021) [19]	To appraise the use of SRs as justification in RCTs …and report the manner in which SRs were incorporated into RCT manuscripts published in those journals.	Cross-sectional	RCTs published in the top four urology journals based on Google Scholar h5 index	USA	November 30, 2014–November 30, 2019	Urology	276 RCTs included. No data on SRs in the ‘Discussion’ section	RCTs published in four high-impact urology journals inconsistently referenced an SR as justification, and 39.1% of our entire sample did not reference an SR at all.
Torgeson T, Evans S, Johnson BS et al. (2020) [25]	To evaluate the use of SRs to justify conducting RCTs in top ophthalmology and optometry journals.	Cross-sectional	RCTs published in the top five Google Scholar h-5 index journals Ophthalmology and Optometry	USA	December 5, 2018	Ophthalmology and optometry	152 RCTs included. 35 (23%) cited SR	Less than one quarter of phase III RCTs cited SRs as justification for conducting the RCT.
Walters C, Torgeson T, Fladie I et al. (2020) [24]	To evaluate whether RCTs … referenced SRs as the basis for conducting a trial.	Cross-sectional	RCTs published in three high-impact factor general medicine journals (NEJM, Lancet, JAMA)	USA	January 1, 2016–August 31, 2018	General medicine	637 RCTs included. No data on SRs in the “Discussion” section	Less than half of the analysed clinical trials cited SRs as the basis for undertaking the trial.

### Risk of bias within studies

Overall, the 15 meta-research studies were rated favourably regarding the risk of bias (see Table 2 for details). Each provided a well-described and unambiguous aim and a match between aim and method. Further, they all considered the same variables in all sources, applied an appropriate method and supported their conclusions with the data. This said, only 1 of 15 meta-research studies presented a protocol [21], and seven meta-research studies [22–24, 27–30] presented no discussion of the limitations of their study. A total of 13 meta-research studies [18, 21–32] were rated as having an unclear risk of bias, as they presented but did not give reasons for their choice of data; three [22, 23, 28] provided poor arguments for choosing variables, and two [26, 31] did not describe the data collection process sufficient. Details are presented in Table 2.

**Table 2:** Risk of bias (high risk of bias, low risk of bias, unclear risk of bias). [For prompts indicating high risk of bias see Additional file 3]

***Study***	***1. Clear and focused aim***	***2. Match between aim and chosen method(s)***	***3. The best data sources(s)***	***4. All important variables considered***	***5. The same variables considered in all sources***	***6. Data collection is transparent, and data unambiguously identified***	***7. Classification of the variables unaffected by prior knowledge about the results***	***8. Appropriate analysis method chosen***	***9. Systematic error considered***	***10. Conclusions supported by data***
Clarke M, Alderson P, Chalmers I. (2002) [27]	Low risk	Low risk	Unclear	Low risk	Low risk	Low risk	High risk	Low risk	High risk	Low risk
Clarke M, Chalmers I. (1998) [18]	Low risk	Low risk	Unclear	Low risk	Low risk	Low risk	High risk	Low risk	Low risk	Low risk
Clarke M, Hopewell S. (2013) [30]	Low risk	Low risk	Unclear	Low risk	Low risk	Low risk	High risk	Low risk	High risk	Low risk
Clarke M, Hopewell S, Chalmers I. (2007) [28]	Low risk	Low risk	Unclear	Unclear	Low risk	Low risk	High risk	Low risk	High risk	Low risk
Clarke M, Hopewell S, Chalmers I. (2010) [29]	Low risk	Low risk	Unclear	Low risk	Low risk	Low risk	High risk	Low risk	High risk	Low risk
Engelking A, Cavar M, Puljak L. (2018) [26]	Low risk	Low risk	Unclear	Low risk	Low risk	Unclear	High risk	Low risk	Low risk	Low risk
Goudie AC et al. (2010) [31]	Low risk	Low risk	Unclear	Low risk	Low risk	Unclear	High risk	Low risk	Low risk	Low risk
Helfer B et al. (2015) [32]	Low risk	Low risk	Unclear	Low risk	Low risk	Low risk	High risk	Low risk	Low risk	Low risk
Hoderlein X, Moseley AM, Elkins MR. (2017) [20]	Low risk	Low risk	Low risk	Low risk	Low risk	Low risk	High risk	Low risk	Low risk	Low risk
Johnson AL, Walters C, Gray, H et al. (2020) [21]	Low risk	Low risk	Unclear	Low risk	Low risk	Low risk	Low risk	Low risk	Low risk	Low risk
Rauh S, Nigro T, Sims M et al. (2020) [22]	Low risk	Low risk	Unclear	Unclear	Low risk	Low risk	High risk	Low risk	High risk	Low risk
Rosenthal R, Bucher HC, Dwan K. (2017) [23]	Low risk	Low risk	Unclear	Unclear	Low risk	Low risk	High risk	Low risk	High risk	Low risk
Shephard S, Wise A, Johnson BS et al. (2021) [19]	Low risk	Low risk	Low risk	Low risk	Low risk	Low risk	High risk	Low risk	Low risk	Low risk
Torgeson T, Evans S, Johnson BS et al. (2020) [25]	Low risk	Low risk	Unclear	Low risk	Low risk	Low risk	High risk	Low risk	Low risk	Low risk
Walters C, Torgeson T, Fladie I et al. (2020) [24]	Low risk	Low risk	Unclear	Low risk	Low risk	Low risk	High risk	Low risk	High risk	Low risk

### Narrative synthesis

Across all the meta-research studies, 635 of the 1724 original studies (36.8%) placed their results in the context of an existing SR in the ‘Discussion’ section. The percentage of original studies using an SR to place their results in the context of existing evidence varied from 9.1% [27] to 48.1% [32]. Progress might be indicated as the number of included original studies is higher in the latest 6 years of the inclusion period, all including 100+ original studies [19–22, 24–26] (Hoderlein et al. [20] Cohort 2), but an equivalent rise in the percentage of original studies placing their results in the context of existing SRs is not demonstrable. Most of the original studies cited or referred to one or more SRs in the ‘Discussion’ section [18, 20, 22, 23, 25–28, 32] (Hoderlein et al. [20] Cohort 2). One of 27 original studies integrated their results within an MA [31], and further 2 of 35 [30], 1 of 29 [29] and 1 of 151 [20] (Hoderlein et al. [20] Cohort 2) explicitly reported integrating results with or updated an existing SR. Five of 1724 original studies (0.29%) integrated their results with the preceding quantitative summation of existing knowledge in the field of interest. Among the subgroup of meta-research studies examining whether the original studies updated an SR [18, 20, 23, 27–31], 5 of 440 original studies did so [20, 29–31] (Hoderlein et al. [20] Cohort 1).

The meta-research studies employed different reporting terms and phrases to assess the *use of SRs to place results in context*. Meta-research studies used terms such as *integrated results* [20, 29–31], *referred to a relevant SR* [18, 23, 26, 27, 32], *stated a comparison, but no further discussion* [31], *cited an SR* [19, 21, 22, 24, 25], *attempted to discuss or explain in relation to other trials* [31] and *summarised some evidence* [20]. We applied the authors’ interpretation straightforward.

Three meta-research studies were not included in the MA, as they did not present sufficient data [19, 21, 24]. All three report the number of citations in the ‘Discussion’ section but not the number of original studies with citations of SRs. Johnson et al. [21] showed citations most prominent in the ‘Discussion’ section compared to the ‘Introduction’ and ‘Methods’ sections in 128 RCTs in three high-ranking orthopaedic journals, while Shepard et al. and Walters et al. [19] could not confirm that result in 276 RCTs in the top four urology journals and 637 RCTs in three high impact-factor general medicine journals [24], respectively.

### Quantitative synthesis

**Fig. 2:**
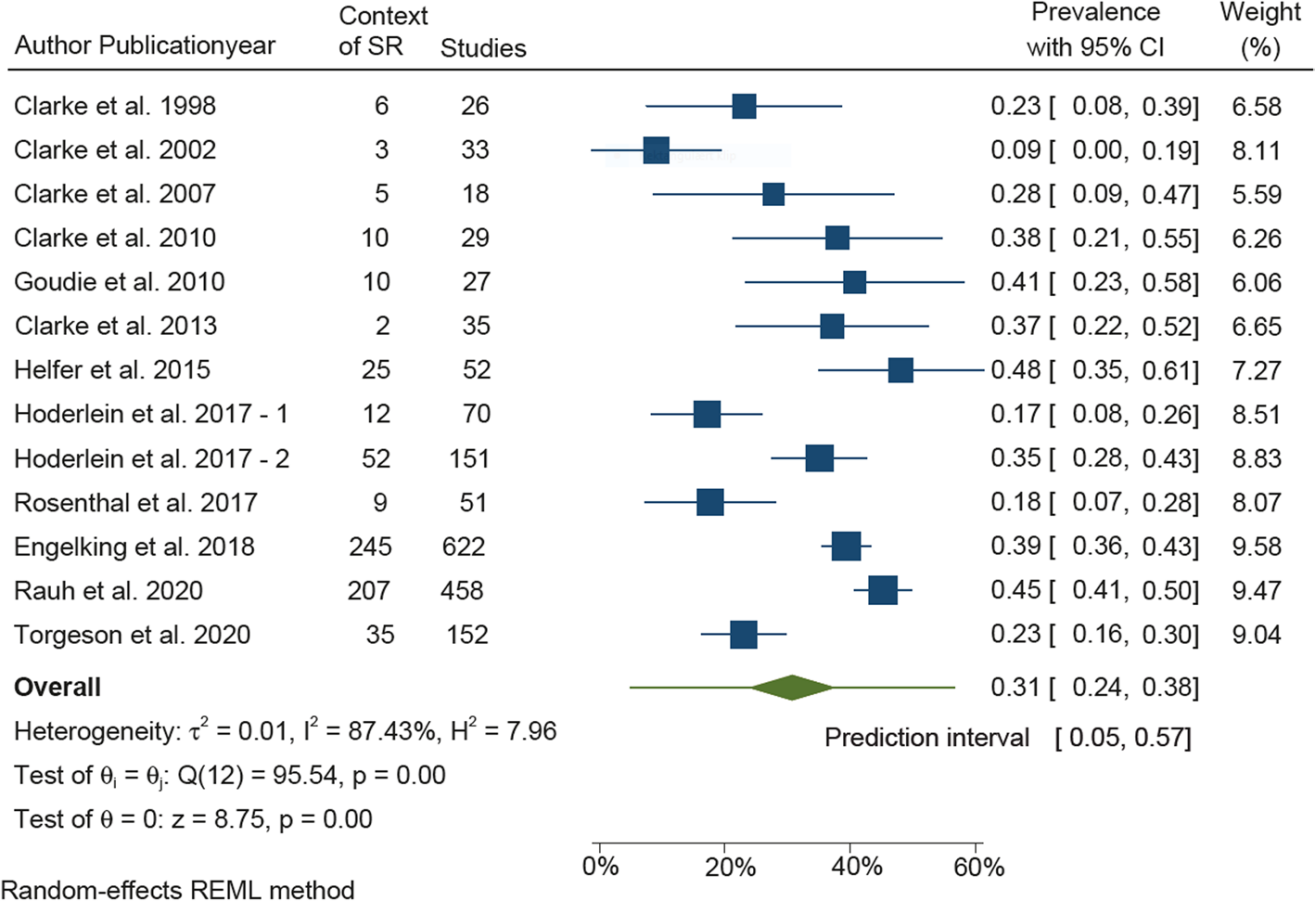
Forest plot prevalence and 95% confidence intervals for the percentage of original studies using an SR when placing results in the context of earlier studies

The total number of meta-research studies in the MA was 12, including Hoderlein et al. [20] presenting two cohorts as seen in Fig. 2. The pooled percentage of original studies included in the meta-research studies presenting data to assess their placing of results in the context of existing evidence (*n* = 13) was 30.7% (95% CI [23.8, 37.6]). Heterogeneity was 87.4%. We conducted an explorative post hoc subgroup analysis, differentiating between studies updating SRs (*n* = 4) and studies citing SRs (*n* = 9), and between the Helfer study (with studies not based on journal publications) (*n* = 1) and the other studies (*n* = 12). These analyses did not explain the heterogeneity, as the between-study variance, tau^2^, increased (data not shown).

The number of original studies placing their results in the context of earlier, similar studies is presented in Fig. 2.

## Discussion

This SR identified and synthesised results from 15 meta-research studies representing 1724 original studies examining if existing studies in clinical research use SRs to place their results in the contexts of earlier studies. On average, approximately one third of the original studies placed their results in context in the ‘Discussion’ section with a mean percentage of 36.8% and ranging from 9.1 to 48.1%. Less than one fifth referred to a *relevant* SR (the term *relevant* was defined by the authors of the included studies). Only one original study [31] integrated their results with existing studies quantitatively with an MA, while four updated an SR [20, 29–31] (Hoderlein et al. [20] Cohort 2). The results display great variation between original studies and during the period of assessment. Even the five meta-research studies with fully identical inclusion criteria but conducted years apart presented fluctuating results [18, 27–30]. Overall, less than half of the original studies contextualised their results with existing evidence, and only a small fraction did so quantitively.

Possible positive progress might appear concerning the prevalence of meta-research studies in the field, as 8 of 15 meta-research studies were published the last 5 years of the 24-yearlong study period [19–26]. Yet, the individual results did not show the progress of more clinical researchers using an evidence-based approach over time, as the percentage of original studies placing the results in context did not differ from the earlier meta-research studies. Additionally, three of the recent meta-research studies [19, 21, 24] did not present any data on SRs in the ‘Discussion’ section. Possible positive progress might also be noticeable in the number of included original studies in each meta-research study, as those including less than 100 original studies were published from 1998 to 2017 [18, 20, 23, 27–32] (Hoderlein et al. [20] Cohort 1), compared to those including more than 100 original studies, which were published from 2015 to 2021 [19–22, 24–26] (Hoderlein et al. [20] Cohort 2). However, this was not succeeded by an increased rate of contextualising, as the rate of citing or updating an SR did not differ between the two groups of meta-research studies. In the same way, the meta-research studies did not display any difference depending on whether the area of interest for the original studies was focused on a specific speciality [19–23, 25, 26, 32] or not [18, 24, 27–31]. Thus, practising EBR in the way of contextualising new clinical results has not improved over time and is not conditional on the number of original studies in the meta-research studies nor the area of interest.

Another noticeable feature of our results is that all meta-research studies were based on original studies published in high-ranking journals, except Hoderlein et al. [20], which was based on studies from a specific physiotherapy database. Some journals, for example, *The Lancet* [10–12], require authors of new studies to place their results in the context of earlier evidence when publishing. Therefore, our results might present an overestimation of contextualising new results in general, as the requirement of contextualising is not standard for all journals.

### Employed definitions of SRs

Our applied definition of the *use of SRs to place results in context* as individually interpreted by the authors naturally has implications for our results. Meta-research studies used terms such as *integrated results* [20, 29–31], *referred to relevant SR* [18, 23, 26, 27, 32], *stated a comparison, but no further discussion* [31], *attempted to discuss or explain in relation to other trials* [31], *cite* [19, 21, 22, 24, 25] and *summarised some evidence* [20]. This broad definition implies that our sample of original studies includes a wide range of studies from those citing an SR to those updating an SR or conducting an MA and is dependent on the authors’ interpretations. The strive for comprehensiveness implies high sensitivity and heterogeneity, and our results must, therefore, be interpreted with caution and as merely representing any original study, *at least mentioning existing knowledge in the field of interests*, but not necessarily contextualising new evidence with existing evidence. Consequently, our results might reflect an overestimation of the extent of contextualising when finishing new studies, if contextualisation implies *relating* new results with earlier, similar results and not just *mentioning*.

It is important to acknowledge that the prevalence of placing new results in the context of earlier results depends on the prevalence of earlier similar studies. One way to control for this might be the authors stating whether earlier similar studies were searched for and located. Eight of the meta-research studies [18, 23, 27–32] reported the number of original studies that ‘claimed to be the first’ original study. However, only three meta-research studies [18, 27, 28] assessed whether this claim was true. Our results maybe have to be moderated by the fact that one cannot assume the prevalence of earlier, existing evidence in all clinical areas.

### Further implications

Our results show pronounced room for improvement when finishing a new study. When results of new clinical studies are not contextualised with existing knowledge in the field, no building up on the knowledge base in a specific clinical area, and thereby no way of establishing whether a new study adds new knowledge or confirmed what we already know [33]. By doing so, it is not possible to establish whether a research question is solved, or further research is needed. This might lead to inefficient use of research funding within a precise clinical question that might already have been answered [5, 34, 35]. Contextualising new results in the way of practising EBR by updating an SR function to prevent redundant research [36].

Furthermore, when not contextualising new results with earlier, similar results, further studies in the area might, therefore, be based on incomplete knowledge in terms of groundings for both the justification and designing of a new study [5, 37–39]. It constitutes a potential bias when only a selective sample of original studies make up the knowledge base in a certain clinical area [13, 36], and it resembles publication bias in terms of not improving the basis on which further studies are based. Practising an EBR approach by automatically updating an SR with results from new studies would improve future clinical studies and potentially increase efficiency in the use of research resources [36]. Although we agree there is an extra workload after finishing an original study when having to update an SR afterwards, which might serve as a barrier to updating [3, 7]. We acknowledge the findings of our study should be validated and placed in the context of other similar studies. However, the comprehensive literature search behind this study, identifying more than 30,000 hits, did not identify a similar study.

### Strengths and limitations

This study was based on a comprehensive search and screening process for meta-research studies and was conducted by a large group of experienced researchers in the field of meta-research. This is a substantial strength, but the literature search was also contingent on the possibility of locating relevant meta-research studies, as no MESH terms exist for *meta-research, EBR,* or similar concepts, which constitute the data in our study. We, therefore, had to be even more meticulous in the second search and used words, phrasing and sentences identified in the first search as key terms in the second search. That left us with a high degree of sensitivity and noise, and we prioritised not missing any relevant studies.

We limited the number of databases in the second search after we tested how many of the already identified relevant studies in the first search could be identified in a MEDLINE and Embase search and whether those not identified in MEDLINE could be identified searching Embase and Scopus. As 47 of 49 tested references were identified in the MEDLINE search and two were identified in Embase and Scopus, we limited the updated search to MEDLINE and Embase.

While the application of a risk of bias checklist created specifically for this study may be seen as a compromise to the risk of bias assessment of the studies, this was a necessary step because no applicable checklist was available. Our checklist was developed based upon other risks of bias tools, including the Cochrane Risk of Bias tool, as well as continuous discussion among six researchers experienced in meta-research, evidence-based medicine, and SR methodology. The reduction in a number of items from 13 to 10 in the first draft of the risk of bias tool, and the omission of reporting quality constitutes the only deviations from the registered protocol and represents an adaptation to this study’s specific aim and setting and displays the profound underlying innovative work behind this study. However, we fully acknowledge that we used a custom-made checklist for these specific meta-research studies, and it needs further elaboration, validation and reliability testing.

Furthermore, the degree of heterogeneity among the studies in the MA calls for elaboration. First, the aims of the meta-research studies varied, and used wording was, for example, *analyse whether existing SRs were mentioned* [26], *make use of previous trial evidence in the reporting* [31] and *discuss new results in light of available evidence* [18, 27–30]. These different study aims naturally have impacted the way each meta-research study was conducted, including the choice of definition of the *use of SRs to place results in context* as presented above. Furthermore, the meta-research studies differ by their selection of included original studies. Although all included RCTs or MAs, some were limited to a specific area of interest, for instance, physiotherapy [20], pharmacological treatment [32] and urology [19], whereas others were based on original studies in general medical journals, for instance, Clarke et al. [18, 27–30], Goudie et al. [31] and Walters et al. [24]. Moreover, one meta-research study was not confined to specific journals but a certain database [20]. In addition, the time of the study period varies from 1998 [18] to 2021 [19]. This timeframe for assessing the degree of contextualisation might be seen as too long—or starting point as too early—as the academic debate around the theme accelerated from about 2005 [12], and the term EBR was introduced in 2011 [38]. Following this line of argument would imply an increase in meta-research studies that place their results in context with earlier, similar studies during the latest year. However, this presumption was not supported by our results.

It is worth noting that 5 of the 15 included meta-research studies had the same first author [18, 27–30] and another five meta-research studies partly had a common group of authors [19, 21, 22, 24, 25]. These two groups of meta-research studies, therefore, represented a large degree of homogeneity compared to the other group and the other five of the included meta-research studies; thus, their priorities had a relatively higher weight in the study material.

The results of this SR are characterised by a high degree of heterogeneity and should be interpreted cautiously. We recommend subgroup analyses when future, similar meta-research studies are conducted. The underlying cause of heterogeneity is not identifiable but could be due to the broad range of clinical health specialities represented among the meta-research studies and methodological features of the meta-research studies. Given the range of clinical specialities, timeframes and differences in approaches to conducting the meta-research studies, the results of this SR cannot be regarded as representative of all clinical trials in health care.

## Conclusion

The findings of this SR display a low rate of placing new results from original clinical studies in the context of existing evidence; on average, only one third of the original clinical studies did so. This illustrates that researchers are failing to use SRs to interpret their new study results within the context of what is already known, thereby not contributing to the accumulation of new evidence on which future studies are based. The results are not promising, especially if our broad and encompassing definition of *placing results in context* is considered. While there is caution in generalising the findings of this SR to all clinical researchers, it does provide evidence that improvement is needed in the application of SRs when placing new results in the context of existing studies. Future efforts should continue to promote the use of an evidence-based approach among clinical researchers and other important stakeholders, such as journals and their editors and reviewers.

## Supplementary Information

**Additional file 1.** Search strategy.

**Additional file 2.** PRISMA-S Checklist.

**Additional file 3.** Risk of bias tool. Prompts for high-risk bias.

**Additional file 4.** List of included studies.

**Additional file 5.** Results of data extraction.

**Additional file 6.** Search protocol – PROSPERO.

## Data Availability

The dataset analysed during the current study is available in Additional files.

## Abbreviations

EBR: Evidence-based research
MA: Meta-analysis
RCT: Randomised controlled trial
REML: Restricted maximum likelihood
SR: Systematic review
