# Supplementary material for: Systematic reviews are rarely used to contextualise new results—a systematic review and meta-analysis of meta-research studies
Source: Syst Rev. 2022 Sep 5;11:189. doi: 10.1186/s13643-022-02062-8 (PMC9446778; doi:10.1186/s13643-022-02062-8)
Supplement: Supplementary file 1 — Additional file 1. Search strategy. [file 13643_2022_2062_MOESM1_ESM.docx]

Additional File 1

Search strategy

“Previous OR Earlier OR Existing” AND “Results OR Evidence OR Trials OR Studies OR Research”. These searches were combined with “Poor OR Biased OR Fraud OR Ignore OR Inadequate OR Selective”. In addition, “Poor OR Biased OR Fraud OR Ignore OR Inadequate OR Selective” was combined with “Citation OR Quotation OR References”. Further, all studies including a “Cumulative Meta-Analysis” was included, and “Poor OR Biased OR Fraud OR Ignore OR Inadequate OR Selective” was also combined with “Systematic Review OR Meta-Analysis”.

More specific searchers were performed and added on to the above-mentioned searches. The first was designed to specifically look for studies evaluating if researchers have used a systematic synthesis of earlier trials when justifying and/or designing their new study. The search terms used was “Previous OR Earlier OR Existing” AND “Results OR Evidence OR Trials OR Studies OR Research” AND “Design OR Introduction OR Background OR Planning OR Conducting OR Research Question OR Justifying”. Further, the search terms “Meta-Analysis OR Systematic Reviews OR Evidence Synthesis” was combined with “Design OR Introduction OR Background OR Planning OR Conducting OR Research Question OR Justifying”.

The search terms used was: “Previous OR Earlier OR Existing” AND “Results OR Evidence OR Trials OR Studies OR Research” AND “Discussion OR Context OR Results”. Further, the search terms “Meta-Analysis OR Systematic Reviews OR Evidence Synthesis” was combined with “Discussion OR Context OR Results”.

# SEARCH JUNE 2015

**Search strings for PubMed Medline, Embase, Cinahl, Web of Science, and Cochrane Methods Studies**

**--------------------------------------------------------------------------------**

**Database: PubMed Medline – June 2015, no language or timespan restriction**

1 (poor quot* OR quotation bias OR poor citat* OR citation bias OR poor citing OR citing bias) in Title/Abstract (752)

2 (redundant research OR redundant publicat* OR duplicate research OR duplicate publicat* OR cumulative meta-analys* OR cumulative research OR research waste) in Title/Abstract (847)

3 (citation network OR research on research OR evidence-based research OR evidence based research) in Title/Abstract (560)

4 (previous OR prior OR earlier OR existing) in Title (35385)

5 (trial* OR research OR evidence OR knowledge) in Title (589052)

6 (publicat* OR published OR citat*) in Title (23858)

7 #4 AND #5 (1907)

8 #4 AND #6 (92)

9 (6) #7 OR #8 (1987)

10 (finding OR identifying OR identification OR determining) in Title (216625)

11 (gaps OR missing OR lack) in Title (37209)

12 (evidence OR knowledge OR research) in Title (404015)

13 #10 AND #11 (399)

14 #10 AND #12 (2331)

15 (12) #13 OR #14 (2665)

16 (systematic review* OR meta-analys* OR evidence) in Title (253157)

17 (design OR planning) in Title (103902)

18 (15) #16 AND #17 (759)

19 #1 OR #2 OR #3 OR #9 OR #15 OR #18 (7541)

20 Research Design [MESH Major topic] (42372)

21 Meta-Analysis as Topic [MESH Major topic] (3563)

22 Review Literature as Topic [MESH Major topic] (3172)

23 #21 OR #22 (6271)

24 #20 AND #23 (3747)

25 (design OR planning) in Title/abstract (906014)

26 (systematic review* OR meta-analys* OR evidence) in Title/abstract (1309600)

27 (trial* OR studies) in Title/abstract (2907191)

28 #25 AND #26 (85393)

29 #25 AND #27 (220930)

30 #28 OR #29 (265682)

30b #30 AND #24 (463)

31(32) Randomized Controlled Trials as Topic [MESH Major topic] (14475)

32(33) Clinical Trials as Topic [MESH Major topic] (42555)

33(34) #31 OR #32 (42555)

34(35) (context) in Title/Abstract (259096)

35(36) #33 AND #34 (1181)

36 editorial policies [MESH Major topic]

37 Publishing/standards [MESH Major topic]

38 ethics committees/standards [MESH Major topic]

39 peer review [MESH Major topic]

40 #36 OR #37 OR #38 OR #39

41 #33 AND #40

42 #19 OR #30 OR #35 OR #41

Addition of Evidence Centre Filters to terms:

SRs

RCTs

**--------------------------------------------------------------------------------**

**Database: Embase OVID – 1974 - June 2015, no language restriction**

1 ((poor adj2 (quot$ OR citat$ OR citing)) OR ((quot$ or citat$ OR citing) adj2 bias$)).tw (77)

2 ((redundan$ adj2 (research OR publicat$)) OR (cumulative adj (meta-analys$ OR research))).tw (574)

3 (citation network OR research on research OR evidence-based research OR evidence based research).tw (803)

4 ((clinical trial$ OR randomi?ed controlled trial$) adj2 (justif$ or valid)).tw (390)

5 (((previous OR prior OR earlier OR existing) adj (research OR evidence OR knowledge OR trials)) AND (publicat$ OR published OR citat$ OR cited)).m_titl (3)

6 ((finding OR identifying OR identification OR determining) adj3 gaps adj3 (evidence OR knowledge OR research)).m_titl (55)

7 ((systematic review$ OR meta-analys$ OR evidence) adj3 (design OR planning) adj3 (new OR future) adj5 (trial$ OR studies)).tw (14)

8 1 OR 2 OR 3 OR 4 OR 5 OR 6 OR 7 (1913)

9 methodology/ (1669524)

10 "systematic review (topic)"/ (11407)

11 "meta analysis (topic)"/ (20387)

12 10 OR 11 (27793)

13 9 AND 12 (2142)

14 (4) ((design$ adj3 new adj3 trial$) OR (identif$ adj2 (gaps OR missing OR lack) adj3 evidence)).tw (626)

15 13 AND 14 (11)

16 "randomized controlled trial (topic)"/ (76229)

17 methodology/ (1669524)

18 16 OR 17 (1740996)

19 ((systematic review OR ((previous OR prior OR earlier OR existing) adj3 trial$)) adj5 context).tw (146) -> (systematic review or context).m_titl.(72811)

20 18 AND 19 (24) (582)

21 "clinical trial (topic)"/ (59051)

22 21 OR 16 (131090)

23 *publishing/ (13104)

24 *professional standard/ (8049)

25 exp "peer review"/ (23496)

26 23 OR 24 OR 25 (43226)

27 22 AND 26 (793)

28 8 OR 15 OR 20 OR 27 (2739)

29 limit 28 to (conference abstract or conference paper or conference proceeding or "conference review")

30 28 NOT 29 (2328)

Addition of Evidence Centre Filters to terms:

SRs

RCTs

**--------------------------------------------------------------------------------**

**Database: Cinahl Ebsco – June 2015, no language or timespan restriction**

1 ((poor N2 (quot* OR citat* OR citing)) OR ((quot* OR citat* OR citing) N2 bias*)) in Title/Abstract (16)

2 ((redundan* N2 (research OR publicat*)) OR (cumulative N (meta-analys* OR research))) in Title/Abstract (33)

3 (citation network) in Title/Abstract OR (evidence-based research OR evidence based research) in Title (627)

4 ((clinical trial* OR randomised controlled trial* OR randomized controlled trial*) N2 (justif* OR valid)) in Title/Abstract (74)

5 (((previous OR prior OR earlier OR existing) N (research OR evidence OR knowledge OR trial*)) AND (publicat* or published or citat* or cited)) in Title/Abstract (3)

6 ((finding OR identifying OR identification OR determining) N3 gaps N3 (evidence OR knowledge OR research)) in Title/Abstract (85)

7 ((systematic review* or meta-analys* or evidence) N3 (design OR planning) N3 (new OR future) N5 (trial* or studies)) in Title/Abstract (13)

8 1 OR 2 OR 3 OR 4 OR 5 OR 6 OR 7 (850)

9 MH study design+ (561766)

10 MH Meta Analysis (16112)

11 MH literature review+ (25958)

12 10 OR 11 (36524)

13 9 AND 12 (12291)

14 ((design* N3 new N3 trial*) OR (identif* N2 (gaps OR missing OR lack) N3 evidence)) in Title/Abstract (129)

15 13 AND 14 (9)

16 research on research in Title/Abstract

17 14 AND 16 (66)

18 MH clinical trials+ (128622)

19 MH Randomized Controlled Trials+ (23172)

20 18 OR 19 (128622)

21 ((systematic review OR ((previous OR prior OR earlier OR existing) N3 trial*)) N5 context) in Title/Abstract (40)

22 20 AND 21 (5)

23 MM publishing (3656)

24 MH "Edit and Review+" (5003)

25 MH Decision Making, Ethical (5295)

26 23 OR 24 OR 25 (13576)

27 26 AND 20 (439)

28 8 OR 15 OR 17 OR 22 OR 27 (1352)

Addition of Evidence Centre Filters to terms:

SRs

RCTs

**--------------------------------------------------------------------------------**

**Database: Web of Science part of the Core Collection – June 2015, no language restriction (I need to update search with proximity operators and check numbers)**

- Science Citation Index Expanded (SCI-EXPANDED) --1900-present
- Social Sciences Citation Index (SSCI) --1956-present
- Arts & Humanities Citation Index (A&HCI) --1975-present

1 TS=((poor NEAR/2 (quot* OR citat* OR citing)) OR ((quot* OR citat* OR citing) NEAR/2 bias*)) (291)

2 (35) TS=(redundan* NEAR/2 (research OR publicat*)) OR TI=(cumulative NEAR/2 (meta-analys* OR research)) OR TI=(duplicate NEAR/2 (research OR publicat*)) OR TI=(research waste) (1158)

3 TI=(citation network OR evidence-based research OR evidence based research) (1105)

4 TI=(research on research) AND TI=((systematic NEAR/1 review*) OR meta-analys* OR evidence) (651)

5 TS=(((clinical NEAR/1 trial*) OR (randomi?ed NEAR/1 controlled NEAR/1 trial*)) NEAR/2 (justif* OR valid)) (372)

6 TS=((previous OR prior OR earlier OR existing) NEAR/3 (trial* OR research OR evidence OR knowledge)) AND TI=(publicat* OR published OR citat* OR cited) (262)

7 TI=((finding OR identifying OR identification OR determining) NEAR/3 (gaps OR missing OR lack) NEAR/3 (evidence OR knowledge OR research)) (101)

8 TS=(((systematic NEAR/1 review*) OR meta-analys* OR evidence) NEAR/3 (design OR planning) NEAR/3 (new OR future) NEAR/5 (trial* OR studies)) (47)

9 #1 OR #2 OR #3 OR #4 OR #5 OR #6 OR #7 OR #8 (3843)

10 TI=(Design Research OR Designs Research OR Research Design OR Research Designs OR Problem Formulation OR Formulation Problem OR Formulations Problem OR Problem Formulations OR Research Proposal OR Proposal Research OR Proposals Research OR Research Proposals OR Scoring Methods OR Method Scoring OR Methods Scoring OR Scoring Method OR Research Techniques OR Research Technique OR Technique Research OR Techniques Research OR Research Technics OR Research Technic OR Technic Research OR Technics Research OR Error Sources OR Error Source OR Source Error OR Sources Error OR Data Quality OR Data Qualities OR Qualities Data OR Quality Data OR Data Reporting OR Reporting Data OR Experimental Design OR Design Experimental OR Designs Experimental OR Experimental Designs OR Matched Groups OR Group Matched OR Groups Matched OR Matched Group OR Research Methodology OR Methodology Research OR Data Adjustment OR Data Adjustments OR Methods OR Study Methodological OR Methodological Study OR Studies Methodological OR Methodological Studies OR Procedures OR Procedure) TS=(7255771) TI=(1059265)

11 TI=(Meta-Analysis OR Meta Analysis OR Data Pooling OR Data Poolings OR Overviews Clinical Trial OR Clinical Trial Overviews OR Clinical Trial Overview OR Overview Clinical Trial) TS=(147409) TI=(53196)

12 TI=(Review Literature OR State-of-the-Art Review OR Review State-of-the-Art OR Reviews State-of-the-Art OR State of the Art Review OR State-of-the-Art Reviews OR State of the Art Reviews) TS=(285941) TI=(73127)

13 #11 OR #12 TS=(419005) TI=(125785)

14 #10 AND #13 TS=(180528) TI=(1318)

15 TS=(clinical trial* OR randomi?ed clinical trial*) (440034)

16 #14 AND #15 (209)

17 TS=(((systematic NEAR/1 review) OR ((previous OR prior OR earlier OR existing) NEAR/3 trial*)) NEAR/5 context) (232)

18 TS=(randomi?ed controlled trial*) (276235)

19 #17 AND #18 (57)

20 TS=(Editorial Policies OR Editorial Policy OR Policies Editorial OR Policy Editorial) (1568)

21 TS=(Ethics Committees OR Committee Ethics OR Committees Ethics OR Ethics Committee) (6390)

22 TS=(Peer Reviews OR Review Peer OR Peer Reviews OR Reviews Peer) (20769)

23 #20 OR #21 OR #22 (28366)

24 TI=(clinical trial* OR randomi?ed clinical trial*) (61131)

25 #24 AND #23 (339)

26 #9 OR #16 OR #19 OR #25 (4437)

27 #26 Refined by: DOCUMENT TYPES: ( ARTICLE OR CORRECTION OR BOOK CHAPTER OR REVIEW OR PROCEEDINGS PAPER OR NOTE OR CORRECTION ADDITION OR REPRINT OR DISCUSSION ) (3272)

**--------------------------------------------------------------------------------**

**Database: Cochrane Methodology Register (CMR, Methods Studies) – June 2015, no language or timespan restriction**

1 ((poor quot* OR quotation bias OR poor citat* OR citation bias OR poor citing OR citing bias) OR (redundant research OR redundant publicat* OR duplicate research OR duplicate publicat* OR cumulative meta-analys* OR cumulative research OR research waste)) in Title/Abstract/Keywords (711)

2 ((clinical trial* OR randomised controlled trial* OR randomized controlled trial*) AND (justif* OR valid)) in Title/Abstract/Keywords (298)

3 (citation network OR evidence-based research OR evidence based research OR research on research) in Record Title (222)

4 (((previous OR prior OR earlier OR existing) AND (trial* OR research OR evidence OR knowledge) AND (publicat* OR published OR citat*)) OR ((finding OR identifying OR identification OR determining) AND (gaps OR missing OR lack) AND (evidence OR knowledge OR research))) in Record Title (15)

5 (((Editorial Policies OR Editorial Policy OR Policies Editorial OR Policy Editorial) OR (Ethics Committees OR Committee Ethics OR Committees Ethics OR Ethics Committee) OR (Peer Reviews OR Review Peer OR Peer Reviews OR Reviews Peer)) AND ((clinical trial* OR randomised controlled trial* OR randomized controlled trial*) OR ((systematic review* OR meta-analys* OR evidence) AND (design OR planning) AND (new OR future) AND (trial* OR studies)))) in Title/Abstract/Keywords (303)

1 OR 2 OR 3 OR 4 OR 5 (1477)

# SEARCH 26 MAY 2021

**Source**: MEDLINE (OVID)

**Time span:** all time (final search limits to 2019 – current)

**Conducted by:** Ane Gjerland, 26^th^ of May 2021

# Part 1: EBR

This part includes all basic EBR terms. There is quite a bit of noise here, but any study using these terms may be relevant; therefore all have been included as they are.

| **Search #** | **Search terms** | **Number of hits (2021)** |
| --- | --- | --- |
|  | evidence-based research.ti,ab. | 943 |
|  | research on research.ti,ab. | 85 |
|  | Meta-research.ti,ab. | 82 |
|  | Metaresearch.ti,ab. | 8 |
|  | cumulative meta-analys*.ti,ab. | 624 |
|  | cumulative metaanalys*.ti,ab. | 6 |
|  | ***1 or 2 or 3 or 4 or 5 or 6*** | ***1741*** |

# Part 2: Redundancy

This part focuses on the topic of redundant research. Terms such as “research waste”, “redundant trials”, etc are gathered in by this search.

The approach here is to combine groups of terms that are typically used in titles and abstracts of studies on redundancy. I have chosen to combine groups of terms instead of combining each possible combination of each term, to simplify the search. The number of hits for each of the large groups of terms (searches #6, #7, and #8) is therefore not important; what is important is the number of hits for the combinations of these terms – the search lines with yellow background.

| **Search #** | **Search name / explanation** | **Search terms** | **Number of hits (2021)** |
| --- | --- | --- | --- |
|  | REDUNDANCY | (redundan* or futil* or (waste* not (wastewater or wastes or waste-to-energy or waste-derived or "waste disposal")) or unnecessary or overlap* or inefficien* or (lack* adj2 novelt*) or ("no new" adj3 knowl*) or "evaluat* identical associations" or "potential duplication" or (("summary effect" or "lack of" or "did not" or "has not") adj3 change*) or ("limited value" adj2 "new") or “value of information analysis”).ti,ab. | 513921 |
|  | STUDY | (study or studies or trial or trials or RCT* or "randomi#ed controlled trial*" or project or projects or report or reports or protocol* or evidence or research or publication* or published).ti,ab. | 13928838 |
|  | REVIEW | ("systematic review*" or meta-analys* or metaanalys* or meta-synthes* or metasynthes* or "evidence synthes*" or "systematic literature search" or "trial sequential analysis" or (chronological adj2 review)).ti,ab. | 316555 |
|  | *(REDUNCANCY ADJ1 STUDY)* | *8 adj1 9* | 1777 |
|  | *(REDUNDANCY ADJ3 REVIEW)* | *8 adj3 10* | 246 |
|  | (STUDY ADJ5 REVIEW) | 9 adj5 10 | 140629 |
|  | *(REVIEW ADJ5 STUDY) ADJ5 REDUNDANCY* | *13 adj5 8* | *157* |
|  | ***(REDUNCANCY ADJ1 STUDY) OR (REDUNDANCY ADJ3 REVIEW) OR (REVIEW ADJ5 STUDY) ADJ5 REDUNDANCY)*** | ***11 or 12 or 14*** | ***2059*** |

# Part 3: Justify

The same approach as for part 2 was tried to gather studies on justification/design/context. However, here the approach unfortunately did not work –too much noise.

So instead, the focus here is on the specific terms used by our already included relevant studies, and made these into search strings. The search strings are therefore very specific, with low numbers of hits, but a high ratio of relevant studies.

| **Search #** | **Search terms** | **Number of hits (2021)** |
| --- | --- | --- |
|  | ("sample size" adj2 citation).ti,ab. | 3 |
|  | ("prior research" adj2 citation).ti,ab. | 3 |
|  | ((avoidable or preventable) adj2 "design limitation*").ti,ab. | 3 |
|  | (question* adj3 "already answered").ti,ab. | 12 |
|  | ((sufficient or satisfactory or acceptable) adj2 "evidence base").ti,ab. | 58 |
|  | ((justifying or justified) adj (study or studies or trial or trials or RCT* or "randomi#ed controlled trial*" or project or projects or report or reports or protocol* or evidence or research or publication* or published)).ti,ab. | 74 |
|  | (("rationale for conducting" or "justification for conducting" or "justif* conducting") adj2 (study or studies or trial or trials or RCT* or "randomi#ed controlled trial*" or project or projects or report or reports or protocol* or evidence or research or publication* or published)).ti,ab. | 64 |
|  | ("inadequate* plan*" adj5 (study or studies or trial or trials or RCT* or "randomi#ed controlled trial*" or project or projects or report or reports or protocol* or evidence or research or publication* or published)).ti,ab. | 8 |
|  | (("use of systematic review*" or "systematic review us*" or "cited systematic review" or "cited a systematic review") adj3 (justif* or rationale or reason or inform or lack* or absence)).ti,ab. | 13 |
|  | ((consider* or "into account") adj1 ((prior or previous or existing or old or older or earlier or established or existing or related or similar) adj (trial or trials or RCT* or "randomi#ed controlled trial*" or protocol* or evidence or publication* or published))).ti,ab. | 52 |
|  | **16 or 17 or 18 or 19 or 20 or 21 or 22 or 23 or 24 or 25** | **282** |

# Part 4: Total

The combination of parts 1-3, and limited to recent studies only.

| **Search #** | **Search terms** | **Number of hits (2021)** |
| --- | --- | --- |
|  | **7 or 15 or 26** | **4055** |
|  | **limit 27 to yr="2019 -Current"** | ***1005*** |

**After EndNote de-duplication: 998**

**After Rayyan de-duplication: 996**
