# Supplementary material for: Systematic reviews are rarely used to contextualise new results—a systematic review and meta-analysis of meta-research studies
Source: Syst Rev. 2022 Sep 5;11:189. doi: 10.1186/s13643-022-02062-8 (PMC9446778; doi:10.1186/s13643-022-02062-8)
Supplement: Supplementary file 3 — Additional file 3. Risk of bias tool. Prompts for high-risk bias. [file 13643_2022_2062_MOESM3_ESM.docx]

Additional File 3

Risk of bias tool

| **Item** | **Prompt for high-risk of bias** |
| --- | --- |
| 1) Is there a clear and focused aim? | A vague or unclear aim of the study |
| 2) Is there a match between the aim and chosen method(s)? | The method chosen will not or is very unlikely to be able to answer the aim of the meta-research study |
| 3) Was the chosen source the best alternative among others? | No or poor argument for selecting the source and/or no or poor description of why other options were not selected |
| 4) Were all important variables considered? | No or poor argument for selecting the variable(s) and/or no or poor description of why other variable(s) were not selected |
| 5) Were the same variables considered in all sources? | Variables used depended upon the source, and/or the same variables were not extracted from all included sources |
| 6) Was the data collection transparent and data unambiguously identified? | No description or poor description of how data were extracted and/or the data extraction were not performed by two independent reviewers |
| 7) Does the classification of the variables/answers appear unaffected by prior knowledge about the results? | No protocol, and/or registration of the background and methods were prepared and made publicly available |
| 8) Was an appropriate analysis method chosen? | The selected analysis(es) does not match the aim and/or was methodologically not correct/widely accepted and/or relevant for the type of data used in the meta-research study, and/or a widely accepted analysis method was not used without any justification |
| 9) Was any possible systematic error or bias taken into consideration in the data collection and/or analysis? | No discussion of the limitations of the study results were included in the Discussion section, and/or the existing limitations/biases had either no impact upon the conclusion, or there was no explanation of why the limitations/biases did not affect the conclusion |
| 10) Is the conclusion supported by the data? | The conclusion and/or parts of the conclusion includes aspects not supported by the results |
