# Supplementary material for: Systematic reviews are rarely used to contextualise new results—a systematic review and meta-analysis of meta-research studies
Source: Syst Rev. 2022 Sep 5;11:189. doi: 10.1186/s13643-022-02062-8 (PMC9446778; doi:10.1186/s13643-022-02062-8)
Supplement: Supplementary file 4 — Additional file 4. List of included studies. [file 13643_2022_2062_MOESM4_ESM.docx]

Additional File 4

List of included studies

Clarke M, Alderson P, Chalmers I. Discussion sections in reports of controlled trials published in general medical journals. JAMA: Journal of the American Medical Association. 2002;287(21):2799-801.

Clarke M, Chalmers I. Discussion sections in reports of controlled trials published in general medical journals: islands in search of continents? JAMA. 1998;280(3):280-2.

Clarke M, Hopewell S. Many reports of randomised trials still don't begin or end with a systematic review of the relevant evidence. Journal of the Bahrain Medical Society. 2013;24(3):145-8.

Clarke M, Hopewell S, Chalmers I. Reports of clinical trials should begin and end with up-to-date systematic reviews of other relevant evidence: a status report. J R Soc Med. 2007; 100(4):187-90.

Clarke M, Hopewell S, Chalmers I. Clinical trials should begin and end with systematic reviews of relevant evidence: 12 years and waiting. Lancet. 2010;376(9734):20-1.

Engelking A, Cavar M, Puljak L. The use of systematic reviews to justify anaesthesiology trials: A meta-epidemiological study. Eur J Pain. 2018;22(10):1844-9.

Goudie AC, Sutton AJ, Jones DR, Donald A. Empirical assessment suggests that existing evidence could be used more fully in designing randomized controlled trials. J Clin Epidemiol.2010.63(9):983-91.

Helfer B, Prosser A, Samara MT, Geddes JR, Cipriani A, Davis JM, et al. Recent meta-analyses neglect previous systematic reviews and meta-analyses about the same topic: a systematic examination. BMC Med. 2015;13:82.

Hoderlein X, Moseley AM, Elkins MR. Citation of prior research has increased in introduction and discussion sections with time: A survey of clinical trials in physiotherapy. Clin Trials. 2017;14(4):372-80.

Johnson AL, Walters C, Gray H, Torgerson T, Checketts JX, Boose M, et al. The use of systematic reviews to justify orthopaedic trauma randomized controlled trials: A cross-sectional analysis. Injury. 2020;51(2):212-7.

Rauh S, Nigro T, Sims M, Vassar M. The use of systematic reviews to justify randomized controlled trials in obstetrics & gynecology publications. European Journal of Obstetrics & Gynecology and Reproductive Biology. 2020;252:627-8.

Rosenthal R, Bucher HC, Dwan K. The Use of Systematic Reviews When Designing and Reporting Surgical Trials. Ann Surg. 2017;265(4):e35-e6.

Shepard S, Wise A, Johnson BS, Sajjadi NB, Hartwell M, Vassar M. Are randomized controlled trials in urology being conducted with justification? Journal of Osteopathic Medicine. 2021;121(8):665-71.

Torgeson T, Evans S, Johnson BS, Vassar M. The use of systematic reviews to justify phase III ophthalmology trials: an analysis. Eye (2020) 34:2041–2047

Walters C, Torgerson T, Fladie I, Clifton A, Meyer C, Vassar M. Are randomized controlled trials being conducted with the right justification? Journal of Evidence-Based Medicine. 2020;13(3):181-2.
