# Supplementary material for: Systematic reviews are rarely used to contextualise new results—a systematic review and meta-analysis of meta-research studies
Source: Syst Rev. 2022 Sep 5;11:189. doi: 10.1186/s13643-022-02062-8 (PMC9446778; doi:10.1186/s13643-022-02062-8)
Supplement: Supplementary file 6 — Additional file 6. Search protocol – PROSPERO. [file 13643_2022_2062_MOESM6_ESM.docx]

Search protocol – PROSPERO

1. ***Review title.***

*The use of systematic reviews when placing new results in context of earlier similar clinical health studies – a systematic review*

1. ***Original language title.***

*(Not applicable)*

1. ***Anticipated or actual start date.***

*01 March 2019 (search)*

1. ***Anticipated completion date.***

*01 March 2021*

1. ***Stage of review at time of this submission.***

*Preliminary searches ticked as started - pilot of the study selection process ticked as started – considerations of ROB guideline ticked as started.*

1. ***Named contact.***

*Associate professor Eva Draborg*

1. ***Named contact email.***

edraborg@sdu.dk

1. ***Named contact address***

*DK - J.B. Winsløws Vej 9B, 5000 Odense C*

1. ***Named contact phone number.***

*+45 21478848*

1. ***Organisational affiliation of the review.***

*Danish Centre for Health Economics, Department of Public Health, SDU in collaboration with The Evidence-Based Research Network*

1. ***Review team members and their organisational affiliations.***
   1. *Associate Professor Jane Andreasen, Department of Physiotherapy and Occupational Therapy, Aalborg University Hospital, Denmark and Public Health and Epidemiology Group, Department of Health, Science and Technology, Aalborg University, Denmark*
   2. *Associate Professor Birgitte Nørgaard, Department of Public Health, University of Southern Denmark*
   3. *Professor, Hans Lund, Centre for Evidence-Based Practice, Western Norway University of Applied Sciences, Bergen, Norway*
   4. *Associate Professor Jennifer Yost M. Louise Fitzpatrick College of Nursing, Villanova University, USA*
   5. *Klara Brunnhuber, Digital Content Services, Elsevier, London, UK*
   6. *Dr. Karen Robinson, Johns Hopkins University School of Medicine, Baltimore, USA*
   7. *Associated Professor Carsten Bogh Juhl, Research Unit for musculoskeletal function and physiotherapy, University of Southern Denmark, and Department of Physiotherapy and Occupational Therapy, University Hospital of Copenhagen, Herlev and Gentofte, Denmark*
2. ***Funding sources/sponsors.***

*No funding*

1. ***Conflicts of interest.***

*No conflicts of interest*

1. ***Collaborators.***
2. ***Review question.***

*The aim of this systematic review is to identify and synthesize results from primary studies evaluating if and how authors of clinical health studies use systematic reviews to place their new results in the context of earlier similar trials by the use of a systematic review.*

1. ***Searches.***

*We will search the following electronic bibliographic databases: MEDLINE (OVID), Embase (OVID), Cochrane Methodology Register. Furthermore, the reference lists of included studies will be checked to identify additional articles and experts’ own literature libraries will be screened. Abstracts from the last five years of Cochrane Colloquium will be screened.

Title / Abstract screening Title / abstract from searches in electronic databases will be screened by two independent reviewers (JA & BN). If one reviewer is in doubt about inclusion or exclusion the reference will be reviewed in full text.

Full-Text screening
Two independent reviewers (JA & BN) will screen the full text. Agreement will be achieved by discussion.
Prior to importation to Rayyan duplicates will be removed in Endnote.*

1. ***URL to search strategy.***
2. ***Condition or domain being studied.***

*The use of systematic reviews when placing new results in context of earlier similar studies by the use of a systematic review.*

1. ***Participants/population.***

*Not applicable*

1. ***Intervention(s), exposure(s).***

*Not applicable*

1. ***Comparator(s)/control.***

*Not applicable*

1. ***Types of study to be included.***

*We will include meta-research studies (or studies performing research on research).*

1. ***Context.***

*Clinical health research.*

1. ***Primary outcome(s).***

*Percentage of primary studies using systematic reviews placing new results in context of existing studies*

*Qualitative analysis of how systematic reviews were used in the placing of new results in context*

*Percentage of studies referring to relevant systematic review(s)*

1. ***Secondary outcome(s).***

Not applicable

1. ***Data extraction (selection and coding).****When all references have been screened following the electronical database search, the reference lists of all included studies will be screened by two independent reviewers (JA & BN).
   Further, the proceedings from the last 5 years of Cochrane Colloquiums will be screened, and possible relevant abstracts will be looked up in electronic databases to find a possible later publication. If new studies (compared to the electronic database search) are added the reference lists of these new included studies will be screened too.*
   *All screening will be performed using the free software Rayyan (https://rayyan.qcri.org/welcome).*

*A standardized form will be developed, piloted, and refined to extract data for study characteristics and outcomes of interest. Two reviewers independently extracted data, with a third reviewer available to resolve conflicts. The following study characteristics will be extracted: information will be extracted from each of the included studies: Bibliographic information; study aims; study design; comparator (if applicable); setting; country; time of data collection; involved professions; results; conclusion; implications for practice/research;*

*Information on outcomes that will be extracted will include:*

- *percentage of primary studies using systematic reviews placing new results in context of existing studies and a qualitative analysis of how systematic reviews were used in the placing of new results in context*
- *Percentage of studies referring to relevant systematic review(s)*

1. ***Risk of bias (quality) assessment.***

*Risk of Bias in SR of EBR related papers*

1. *Internal validity*
2. *Clear and focused aim*
3. *Good match between aim and chosen method(s)*
4. *Was potential confounding factors identified and taking into consideration if there was?*
5. *Was the chosen source the best alternative among others? (Selection bias)*
6. *Was the variable(s) chosen (A) the most important, (B) same for all included sources?*
7. *Was important variables neglected?*
8. *Did the data collection relied on subjective evaluation or was it unambiguous data that could be identified explicitly?*
9. *Could the classification of the variables / answers have been affected of a prior knowledge about the results?*
10. *Was an appropriate analysis method chosen?*
11. *Was there missing data?*
12. *Was any possible systematic error or biases taken into consideration in the data collection and/or analysis?*
13. *Conclusion supported by the data*

*Reporting quality*

1. *Was there a well described and unambiguous aim?*
2. *Was the methods well described?*
3. *Was all relevant results reported?*
4. *Was there a list of included studies (sources) (in text or appendix)?*
5. *If studies (sources) was excluded, was the exclusion acceptable explained?*
6. *Was all phases of the methods transparently described?*
7. ***Strategy for data synthesis***

*We assume that this will be a configurative review, but if aggregated data emerge, meta-analysis will be conducted. Even though aggregated data should be accessible, a narrative analysis might be chosen dependent on the clinical and statistical heterogeneity of the included studies.*

1. ***Analysis of subgroups or subsets.***

*No planned subgroup analysis*

1. ***Type and method of review.***

*The information required here relates to the topic and outcome of the systematic review.*

1. ***Language.****English*
2. ***Country.***

*Denmark*

1. ***Other registration details.***

*EBR Network Road Map number 201754*

1. ***Reference and/or URL for published protocol.***

*None*

1. ***Dissemination plans.***

*Dissemination plans will include: a paper will be submitted to a leading journal in this field, presentations at relevant conferences, Twitter, posting of findings on EBRNetwork website.*

1. ***Keywords.***

*Systematic review; context; evidence based research; EBR*

1. ***Details of any existing review of the same topic by the same authors.***
2. ***Current review status.***

*Please, see #5*

1. ***Any additional information.***

*This review is carried out as a project of the Evidence based Network (ebrnetwork.org)*

1. ***Details of final report/publication(s).***
